# Vibration Serviceability of the Aberfeldy Footbridge under Various Human-Induced Loadings

**DOI:** 10.3390/ma16072890

**Published:** 2023-04-05

**Authors:** Izabela Joanna Drygala, Joanna Maria Dulińska, Nicola Nisticò

**Affiliations:** 1 Faculty of Civil Engineering, Cracow University of Technology, 31-155 Cracow, Poland; 2 Department of Structural and Geotechnical Engineering, Sapienza University of Rome, 00184 Rome, Italy

**Keywords:** footbridges, composite material, numerical modeling, innovative applications, dynamic analysis, vibration comfort criteria assessment, glass-fiber-reinforced polymer (GFRP), elastic and mechanical analysis

## Abstract

Developing new structural materials, such as composite materials, has provided many opportunities in bridge engineering. Among these materials, glass-fiber-reinforced polymers (GFRPs), in particular, have found applications in footbridges. However, some of the commonly recognized advantages of GFRPs, such as the high values of the strength/weight ratio, can also be considered disadvantageous for certain realizations, particularly when the composite material used in a footbridge is, for example, subjected to dynamic actions such as those that are induced by wind and walking and/or running users. The induced accelerations can reach high values in comparison to recommended thresholds. Further, the natural frequency decays during the service life, reducing the capacity of the frequencies to move toward the frequency content of the pedestrian step. In this framework, the presented research is devoted to the dynamic comfort assessment of a pioneering cable-stayed GFRP pedestrian bridge, Aberfeldy, which was assembled in 1992 in the eponymous small town, which is located in Scotland (UK). The assessment was numerically performed through a finite element (FE) model, which was tuned based on the literature data concerning geometry, structural details, and in situ-acquired frequencies. The analyses carried out in this study include the evaluation of the accelerations’ time histories, which were induced when simulating a set of pedestrian path scenarios, and the dynamic actions that occur during pedestrian traveling. Specifically, different values of velocity and step frequency were considered as well as the inclusion of walking and running movements. Then, based on the acceleration values, the assessments of comfort criteria for the current standards were elaborated while also recognizing that the peak accelerations—usually attained for short periods—cannot be the only parameters considered in evaluating the pedestrian bridge capacity. This investigation allowed a dynamic comfort rating to be established for the Aberfeldy footbridge.

## 1. Introduction

Composite FRP materials were, and still are, extensively used for the retrofitting of existing structures [1,2,3,4,5]. As far as new constructions are concerned, FRPs are currently implemented in various limited situations that mainly include footbridges [6,7], as reported in [8], where Alper et al. (1977) presented a reinforced plastic pedestrian bridge, a bridge erected (1972) in Tel Aviv (Israel) that can be mentioned among the relevant pioneering applications. Further references to existing applications can be found in [5], [9], and in [10], where the preliminary design of an FRP cable-stayed pedestrian bridge was presented.

As far as vehicular bridges are concerned, composite structures that combine concrete slabs with either CFRP [11,12] or GFRP [13] sections are preferred. In the case of pedestrian bridges, GFRP material [14,15,16,17,18,19,20] is preferred, mainly due to the lower cost of glass when compared with carbon fibers.

Pedestrian bridges that have been realized and studied in the past highlight certain deficiencies in terms of pedestrian-induced vibrations. The use of FRP does not solve this problem [21,22]; on the contrary, this issue is only emphasized when specific studies have not been carried out during the design process. As a result, it is known that (1) FRP bridges cannot be designed by only considering the frequencies over the thresholds that are suggested by the recommendations for conventional structures [23]; (2) pedestrian steps should be properly simulated, which includes studying higher harmonics [24]; and (3) tuned mass dampers (TMD) can be adopted to reduce induced accelerations [25].

Since GFRP materials were implemented as a structural tissue of footbridges, certain technical problems in terms of maintenance and durability have occurred. Furthermore, it was reported that the dynamic performance of composite material footbridges could be insufficient for the comfort of potential users [21,22,23,24,25]. Hence, studies oriented toward constructing a definition of comfort criteria for FRP pedestrian bridges are needed in order to integrate the general remarks that are included in the standards and guidelines.

Among the past documented studies, those presented in [21,22] can be mentioned; however, more studies are needed to draw specific guidelines, especially for cable-stayed bridges for which preliminary studies have been presented in [10], where a full GFRP pedestrian bridge was discussed by one of the authors of this work: (1) The main issues to be solved concerned the wind and pedestrian effects; (2) wind tunnel tests were scheduled, and (3) numerical studies were carried out in order to simulate the pedestrian effects on existing, similar pedestrian bridges.

The oldest, and still used, fully plastic-made pedestrian bridge was assembled in 1992 in Aberfeldy (Scotland, UK). The Aberfeldy footbridge was closed for over two years due to safety fears. After retrofitting, the structure was again open for use in 2018. Even if other composite material footbridges are still being assembled and used, the Aberfeldy footbridge is a structure that draws researchers’ attention as a potential case study in terms of illustrating the total life cycle for a composite material footbridge. [26,27,28,29,30,31]. However, to the authors’ knowledge, the multivariant assessment of the Aberfeldy footbridge, under various human-induced loadings and in terms of contemporary design standards, could be introduced as a contribution to the current state-of-the-art knowledge, specifically in the field of composite material footbridges.

The presented framework stimulates cooperation to study the pioneering Aberfeldy pedestrian bridge [26,27,28,29,30,31]. The obtained results will be presented in the following sections and will be organized as follows: (1) the materials and methods are introduced first; (2) the morphology and finite element (FE) model of the Aberfeldy footbridge is then specified; (3) based on the numerical modal model, the resonance risk level is quantified; (4) the pedestrian-induced dynamic loads are then classified based on the assumed frequency and structural eigenpairs; and (5) the comfort criteria assessment are checked against the current standards.

## 2. Aberfeldy Footbridge

**Figure 1:**
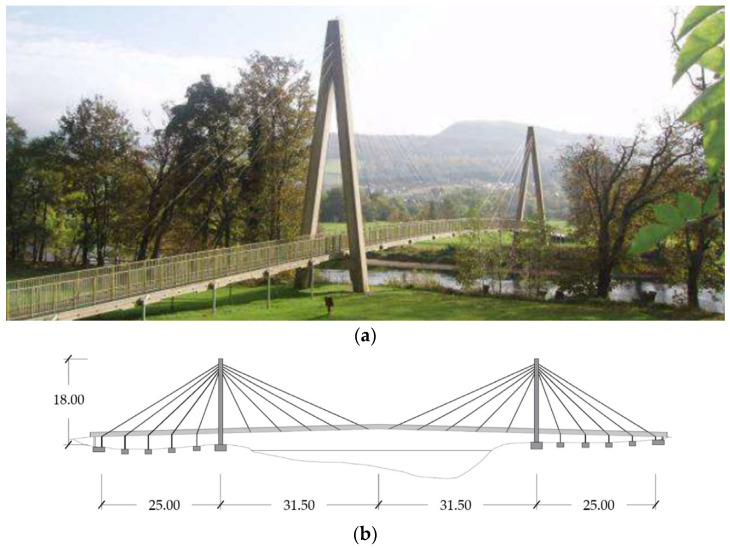
Aberfeldy footbridge: (**a**) the general view [29] and (**b**) the longitudinal section (dimensions in m).

The cable-stayed FRP footbridge (Figure 1a) is located over the Tay River in Aberfeldy, Scotland (United Kingdom). It was erected in 1992 as part of the infrastructure that supports the Aberfeldy Golf Club. The bridge was studied in the past [26,27,28,29,30,31] to acquire information on details, materials, frequencies, and damping: investigations were essentially scheduled to check the status of the structural elements at the time.

### 2.1. Structural Layout

**Figure 2:**
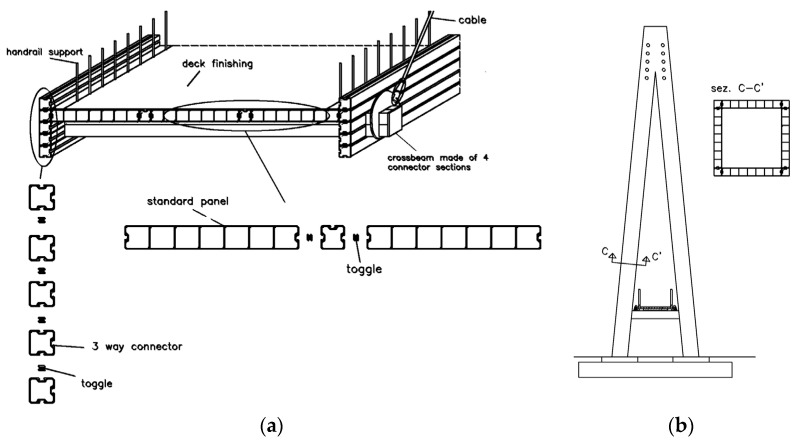
Details of the footbridge: (**a**) the cross-section and (**b**) the pylon.

The bridge in question, having (Figure 1b) a 63.00 m long central span and two side spans of 25.00 m, was conceived and realized by assembling GFRP elements, except for aluminum connections and reinforced concrete foundations. The deck (Figure 2a) and pylons (Figure 2b) were realized [28] using cellular elements (80×80 mm), which were preliminarily jointed through toggle and dog bone connections that are bonded with epoxy adhesive. The Parafil [32,33] stay cables, which connect the pylons with the deck through an aluminum spike [34] anchorage, consist of dry aramid and are compacted in polymeric sheets.

The deck is supported by a system of transversal and longitudinal cellular beams (Figure 2a), whereby the latter hosts the aluminum elements to which the cables are connected. Along the spans of the deck, some of the longitudinal plank cells are filled with ballast material to weigh down the deck, thus preventing it from being lifted under severe wind conditions. The total FRP weight is ≈185 kN.

### 2.2. Frequencies and Damping Properties 

Aberfeldy was, in the past, monitored in the framework of a maintenance program. As far as the knowledge of the authors is concerned, in 1995, 2000, and 2019, the frequencies and related damping ratios were evaluated [26,27,31] based on the in situ-recorded accelerations. Those values are reported in Table 1.

## 3. Materials and Methods

In general, the dynamic behavior and, consequently, the comfort level of walkways depends on three key issues: (1) The modal parameters of the structure (natural frequency, mode shapes, and damping ratio); (2) the excitation source (type of loading induced by the users of the walkways); (3) the footbridge user response (human perception of vibrations). Since excitation sources and the human perception of vibration are not modifiable, the modal parameters of footbridges have become a principal aspect to consider.

Hence, as far as the induced pedestrian vibrations are concerned, the design procedure requires the following: (1) the definition of conventional thresholds that are aimed toward a reduction in the resonance risk; (2) a simulation of the path that would be taken by people on the bridge through a mathematical model; (3) the prediction of the path scenarios, which include walking and running pedestrians; (4) the calibration of an FEM model to correctly reproduce the modal parameters in terms of modal shapes, frequencies, and damping ratios.

### 3.1. The Resonance Risk Evaluation

**Figure 3:**
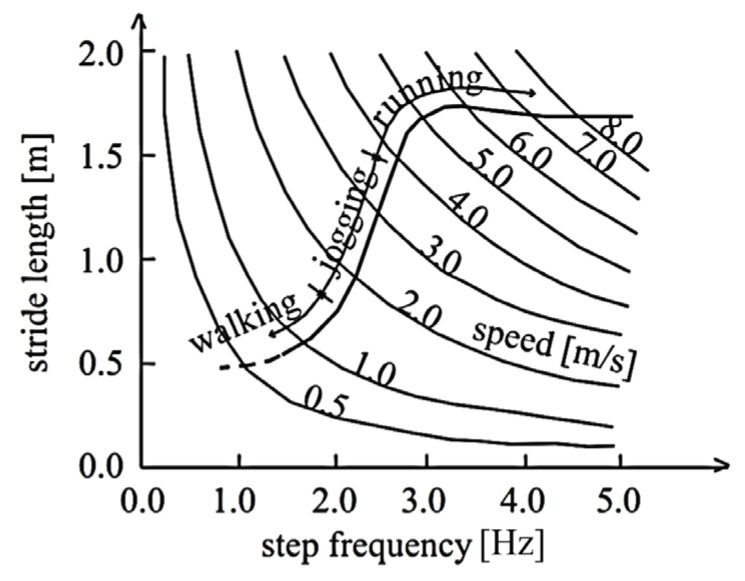
Frequency, velocity, and stride length range for walking, jogging, and running [35,36].

Movement, when periodic, is characterized by the following: velocity ($v_{u}$), step length ($l_{u}$), and step frequencies ($f_{u}=\frac{v_{u}}{l_{u}}$). It is usually classified as walking, jogging, and running. For each of these conditions, the reasonable values of those parameters ($f_{u}$,${\;v}_{u}$, and $l_{u}$) can be defined as reported in [35,36], which are in terms of either discrete values (see Table 2) or, given a speed velocity, the dependence of stride length on step frequency, and can be defined as reported in Figure 3.

In noticing that the superior limit for step frequency (Figure 3) is 5 Hz, it can be assumed that the risk of resonance is negligible if the lower structural frequency ($f_{s\_\mathrm{lower}}$) is greater than 5 Hz. Further, given the values of $f_{s\_\mathrm{lower}}$, the following cases can be considered: the risk of resonance (a) can be excluded if $f_{u}$ is lower than $f_{s\_\mathrm{lower}}$; however, (b) cannot be excluded if $5\;\mathrm{Hz}>f_{u}>f_{s\_\mathrm{lower}}$ since the resonance can be triggered for a higher structural frequency. Given that the risk of resonance depends on the structural frequencies, (1) the EC 1990:2002 (A1:2005) [37] suggests that a sufficient level of pedestrian comfort is accomplished if the structural frequencies are greater than 5.0 Hz for the vertical direction and 2.5 Hz for the horizontal and torsional modes, and (2) the SÉTRA recommendations [38] provide the levels of resonance risk (see Table 3) as a function of the structural frequencies.

However, it is worth noting that those thresholds are not to be considered as the reference values [23,24] for FRP footbridges: it follows that the risk of resonance has to also be evaluated for the fundamental frequencies, which could be excited by harmonics having frequencies that are higher than 5 and 2.5 Hz for the vertical and lateral stepping, respectively. With these premises, the studies presented here are based on dynamic numerical analyses that simulate the pedestrian steps through literature models and consider different configurations of pedestrian-induced loadings during walking or running activities, where a set of load cases were combined to capture the most demanding configurations.

### 3.2. Human Walking and Running: Equivalent Forces 

**Figure 4:**
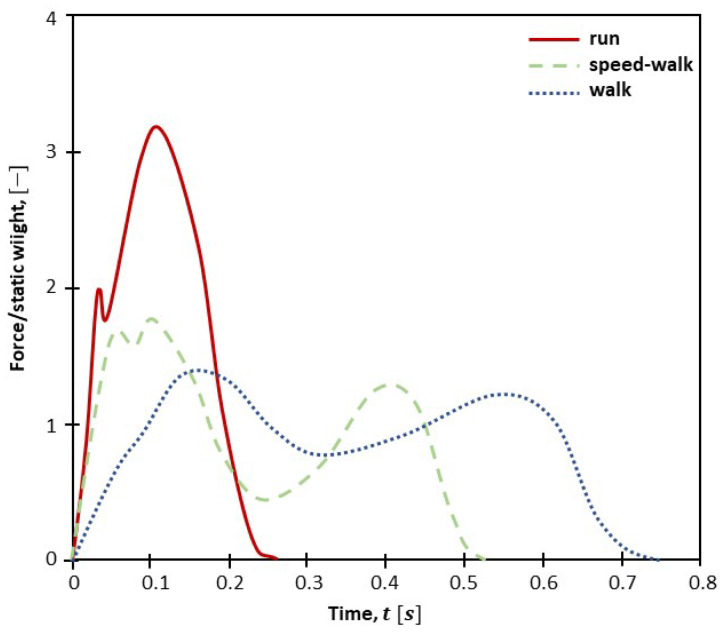
Normalized vertical contact force against time: run, speed-walk, and walk conditions. An elaboration of [39].

**Figure 5:**
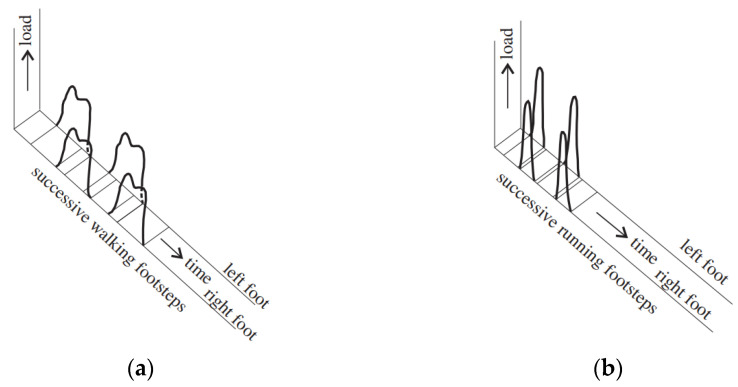
A 3D vertical time history of the ground force reactions (GFR) for the left and right feet during (**a**) walking and (**b**) running [35].

Movement implies that the transmitted forces depend on leg position as well as the stance; the pace, then, is the time when the feet are in contact with the ground. The pace time and forces, in turn, depend on step velocity and body position. However, this is different for walking, jogging, and running. Assuming that jogging can be considered a faster walk, typical normalized forces are reported in Figure 4 that, as discussed in [39], denote the following: (1) When the velocity increases, the peak force increases and the pace time decreases (it can also be assumed that the global transmitted force is independent of the type of movement); (2) walking, which is different to running, denotes two peaks that occur when the heal (first peak) and the toe push on the ground (after the first peak the force decreases, and this is due to the body lift). It is worth noting that (Figure 5) the rear and front foot are (1) differently placed in the space, and (2) depending on the type of motion, can act simultaneously during a time interval, which is included in the force phase.

The step evolution in time, as reported in Figure 5, can be approximated through the (k) harmonic series. In addition, the mathematical model proposed by Bachman [40,41] can be applied to longitudinal (X), lateral (Y), and vertical (Z) forces. The Bachman model is recommended by the ISO 10137:2007(E) standard [42] and the SÉTRA document [38]. Hence, for the walking and running cases, the force in time (t) can be represented by the Fourier series reported in Equations (1)–(3). The single term of the nth harmonic depends on the pedestrian weight G, which is assumed equal for each harmonic, the frequency $f_{n}$, the phase angle $\varphi_{n}$, and $\alpha_{n}$, the dynamic load factor (DLF). Based on the experimental results, the previously defined parameters have been proposed by Zivanovic and Pavic [36]:

(1) $$F_{X}\left(t\right)=G\overset{k}{\underset{n=1}{\sum }}\alpha_{n}^{X}\sin\left(\pi nft+\varphi_{n}^{X}\right)$$

(2) $$F_{Y}\left(t\right)=G\overset{k}{\underset{n=1}{\sum }}\alpha_{n}^{Y}\sin\left(\pi nft+\varphi_{n}^{Y}\right)$$

(3) $$F_{Z}\left(t\right)=G(1+\overset{k}{\underset{n=1}{\sum }}\alpha_{n}^{Z}\sin\left(2\pi nft+\varphi_{n}^{Z}\right))$$

**Figure 6:**
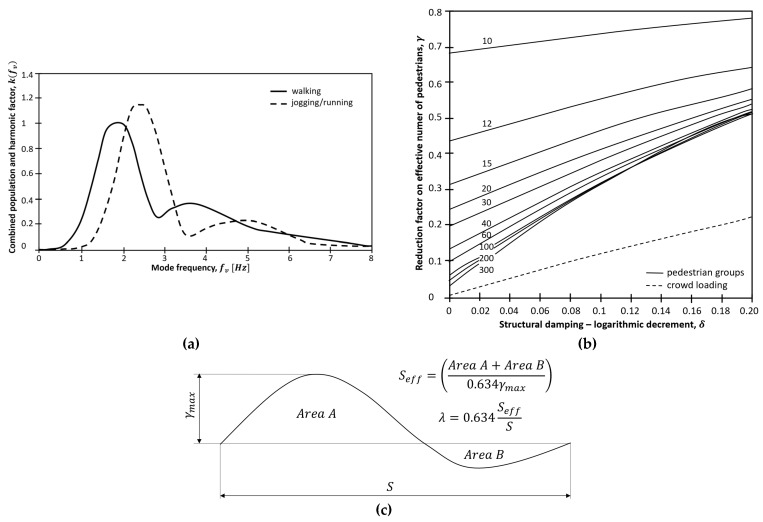
The vertical pulsating force [43]. (**a**) The harmonic factor $\left(k\right)$; (**b**) the reduction factor of the effective number of pedestrians $\left(\gamma \right)$ regarding the dependence on damping (δ) and the effective span length ($S_{\mathrm{eff}}\;m$); and (**c**) the effective span length $\left(S_{\mathrm{eff}}\right)$.

A further simplified model is provided in BS EN 1991-2:2003 (UK National Annex to EC 1) [43]. The vertical pulsating force, evaluated according to Equation (4), depends on the number of pedestrians N, the reference amplitude of the applied fluctuating force $F_{0}$, the considered natural frequency Hz of the vertical mode $f_{v}$, a factor $k\left(f_{v}\right)$ (Figure 6a) that is introduced to include the dependence on the considered frequency, and γ, a factor that includes the dependence on damping (Figure 6b) and the effective span length, $S_{\mathrm{eff}}$—which is evaluated, as reported in Figure 6c.

(4) $$F\left(t\right)=F_{0}k\left(f_{v}\right)\sqrt{1+\gamma \left(N-1\right)}\sin\left(2\pi f_{v}t\right)$$

As far as runners are concerned, the ‘half-sine’ model [44,45,46] that is reported in Equation (5) can be adopted:

(5) $$F\left(t\right)=\left\{\begin{array}{lll} A_{r}G_{0}\sin\left(\frac{\pi f}{k}t\right) & for & \;\;j\cdot T_{r}<t\leq \left(j+k\right)T_{r}\\ \;\;\;0 & for & \;\;\;\left(j+k\right)\cdot T_{r}<t\leq \left(j+1\right)T_{r} \end{array}\right.$$

where k is the contact time factor ($k=t_{\mathrm{cr}}/T_{r})$; $A_{r}$ is the dynamic impact factor ($A_{r}=\pi /\left(2\cdot k\right)$); $t_{\mathrm{cr}}$ is the pace time; $T_{r}$ is the period of running ($T_{r}=1/f$); t is the time; and j=0,1,2, …,n.

### 3.3. Human Traveling: The FEM Model and Path Scenarios

**Figure 7:**
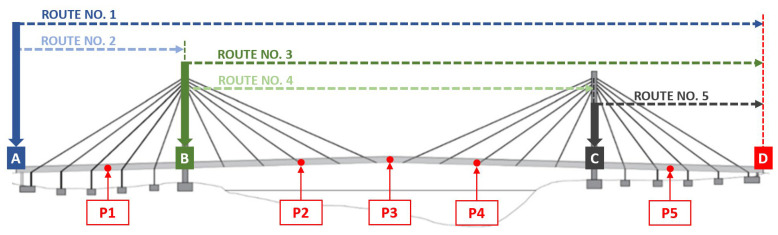
Dynamic simulations: the considered cases and the points (P1–P5) selected along the central alignment to evaluate the induced accelerations.

Assuming that the crowd condition can be considered a static load, the scenarios (see Figure 7) consist of routes that a pedestrian uses to run or walk across the bridge. Moreover, the four considered routes have been further combined (see Table 4) to reproduce the worst-case condition for each span. The pedestrian-induced forces were simulated through Equations (1)–(5): for each path, (1) different force frequencies were considered; (2) each frequency was considered equal to a selected structural frequency to impose the resonant conditions; and (3) when more routes were considered, in a single path, the pedestrian-induced forces of each route were considered in phase or counterphase, as in the cases of paths VI and VII (see Table 4). 

**Figure 8:**
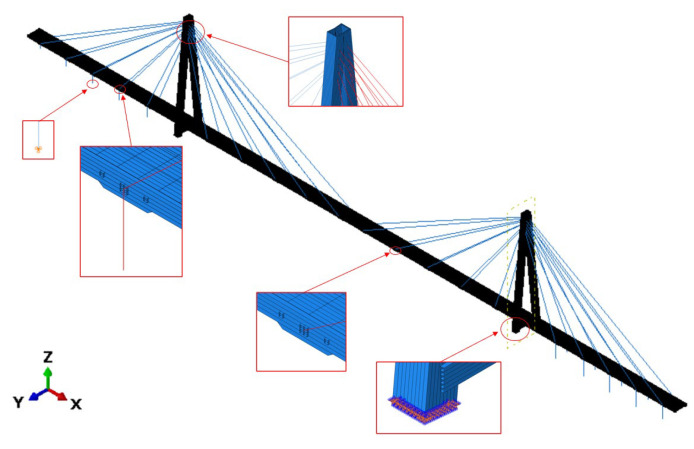
The FE model of the cable-stayed composite material footbridge.

The FEM model of the footbridge (Figure 8), implemented in ABAQUS/Standard [47], is assembled through linear S4R and T3D2 elements, the total number of which is 113,084. The cables were discretized through the frame elements, having an equivalent Young’s modulus. Meanwhile, the shell orthotropic elements were used to model the pylon and deck elements. For this, the lamina theory [48] was assumed, and, for each layer, the reduced lamina stiffness ($Q_{\mathrm{ij}}$) was evaluated, as reported in Equations (6)–(8), where (1) $E_{11},{\;E}_{22}$ are the Young’s Moduli, which are along and orthogonal to the prevalent fiber direction; (2) $\nu_{21}=\nu_{12}\frac{{\;E}_{22}}{{\;E}_{11}}$ are the Poisson’s coefficients; and (3) $G_{12}$ is the shear moduli:

(6) $$Q_{11}=\frac{E_{11}}{1-\nu_{12}\nu_{21}}$$

(7) $$Q_{22}=Q_{11}\frac{E_{22}}{E_{11}}$$

(8) $$Q_{33}=G_{12}$$

For each case, the damping ratios were assumed as frequency dependent and in agreement with the experimental values that were evaluated in 2019 (Table 1). Further, the sensitivity of the dynamic response to the footbridge damping ratios was examined. Given the different scenarios, the induced accelerations are recorded in the five points that are reported in Figure 7.

### 3.4. Comfort Criteria Assessment

When conducting the footbridge assessment, the induced accelerations, $a\left(t\right)$, are usually assumed in order to define the comfort level. This is usually performed as per the following steps: (1) the peak absolute value (PAV); alternatively, (2) the root-mean-square (RMS) value can be considered (Equation (9)) when evaluating this between the time intervals ($t_{1},{\;t}_{2}$) that generally include the PAV.

(9) $$RMS={\left[\int_{t_{1}}^{t_{1}+∆t}{\left[a\left(t\right)\right]}^{2}dt\right]}^{\frac{1}{2}}$$

Depending on these values, the comfort level can be defined based on acceptable values. In addition, these values, depending on the assumed recommendation, are ruled as a function of the frequency content of $a\left(t\right)$, whereby it is given that the level of perceptibility depends on the frequency ranges.

If the PAV is assumed, Eurocode 1 [35], SÉTRA [36], and the UK National Annex to Eurocode 1 [43] can be adopted. SÉTRA [36] defines four ranges, which are different for the vertical and horizontal accelerations, and each range is classified, as reported in Table 5.

According to Eurocode 1 [37], the acceptable values are $0.7\;\frac{m}{s^{2}}$ for the vertical direction, 0.2 for the horizontal condition and normal use, and $0.4\;\frac{m}{s^{2}}$ for exceptional crowd conditions. The UK National Annex to Eurocode 1 [43] provides a threshold that depends, according to Equation (10), on four parameters ($k_{1},k_{2},k_{3},k_{4})$, the values of which (Table 6) were defined for the Aberfeldy footbridge and based on the site usage, the routing redundancy, the height of the structure, and the exposure. The values obtained through Equation (8) have to be higher than $0.5\;\frac{m}{s^{2}}$ and lower than $2.0\;\frac{m}{s^{2}}$.

(10) $$a_{limit}=1.0*k_{1}*k_{2}*k_{3}*k_{4}\;\frac{m}{s^{2}}$$

**Figure 9:**
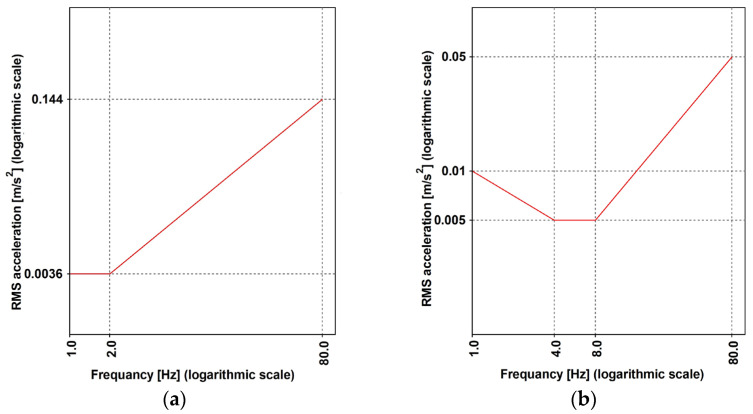
The vibration base curves for the RMS acceleration (ISO [42]): (**a**) the side-to-side and back-to-chest direction; and (**b**) the foot-to-head direction.

The ISO standard [42] chooses RMS as the parameter, thereby suggesting an evaluation according to Equation (9), where ∆t=1 s is assumed. The thresholds are defined as a function of the frequency. In addition, the direction of the acceleration, according to a referral system, is local to the human body. The considered directions are side-to-side, back-to-chest, and foot-to-head directions, and the pedestrian can be considered a walker or a receiver when they are standing. The recommended values for the RMS parameter are reported in Figure 9, whereby those values have to be scaled through the factor that is given in Table 7. The design situations are detailed as per the following: (1) one pedestrian walking and one or more users standing on the footbridge slabs, thereby acting as the receivers; (2) the average user traffic, which refers to a daily occurrence rate; (3) larger groups of user attendance (i.e., more than 15 users); and (4) the occasional events or festivals (where applicable).

## 4. Results and Discussion

**Figure 10:**
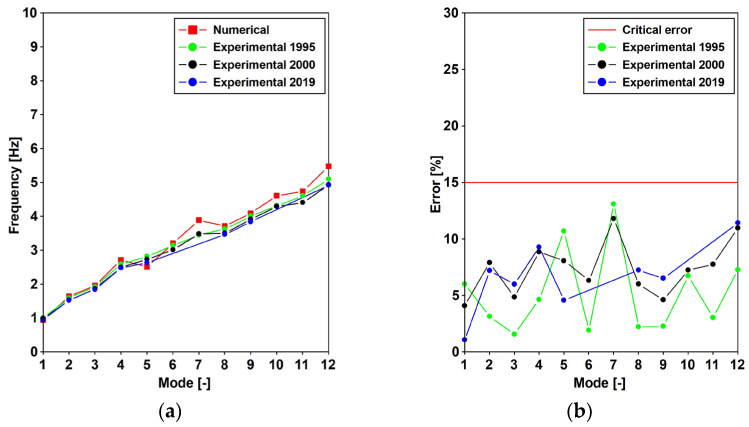
The modal frequencies [Hz]: the numerical vs. the experimental values, and the percentage error.

The maintenance program scheduled for Aberfeldy allowed (Table 1) the experimental evaluation of the main frequencies and the related critical damping ratios. The data were adopted in order to tune the numerical model (Figure 10) that served to simulate the effect of pedestrians traveling, in terms of the vertical and horizontal forces. Depending on the investigations that were carried out, the assumed force model was selected among those reported in Equations (1)–(5). The adopted material and structural eigenpairs are presented in the following section. Then, the discussion regarding the comfort level assessment will be detailed.

### 4.1. Modal Parameters of the Footbridge

Preliminarily, the model and its mechanical properties were calibrated to minimize the errors between the values of the numerical and experimental frequencies: (1) the obtained material properties, adopted to evaluate the reduced lamina stiffness (Equations (6)–(8)), are reported in Table 8; (2) the cable Young’s modulus was assumed to be equal to 126.50 GPa for the Parafil Type G (Kevlar 49 core fibers) material [32,33].

**Figure 11:**
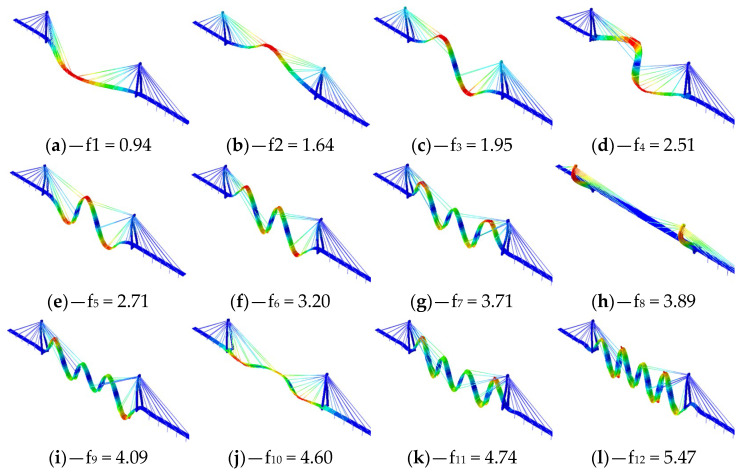
The numerical simulations: the mode shapes and frequencies [Hz] (the color spectrum that was adopted for the orthonormalized vectors—red expresses the maximum value and blue expresses the minimum value).

Further, for each mode, the obtained frequency was compared with the experimental values that were obtained in 1995 [26], 2000 [27], and 2019 [31], which have already been reported in Table 1 and are proposed in Figure 10a. Furthermore, the maximum percentage error that resulted was equal to 13%, while the average that resulted was lower than 7% (Figure 10b). The modal shapes and frequencies are reported in Figure 11.

### 4.2. Pedestrian-Induced Accelerations vs. Structural Performance

When considering the first vertical and the second horizontal modes, the obtained experimental values (Table 1) range between 1.52–1.59 and 0.95–1.00 Hz, and the numerical values are 1.64 and 0.94 Hz. It follows, then, that those values (a) cannot be considered acceptable according to Eurocode 1 [35], which suggests thresholds of 5.0 Hz for the vertical direction and 2.5 Hz for the horizontal direction, and (b) highlights the medium/maximum risk level (Table 3)—thus adopting the classification that is suggested by SÉTRA [38]. Consequently, dynamic simulations are needed and have been carried out, as described.

### 4.3. Pedestrian Frequency Selection

Runnability analyses are usually finalized in the evaluation of the effects that result from pedestrian movements, where the n values of the frequency step are selected and analysis is carried out for each. It is worth noticing that, generally, each of the n-selected values needs to be equal to a structural frequency. It follows then, that depending on the considered movement (i.e., running or walking) and based on the data reported in Figure 3, a range of realistic frequencies and the consequent step length and velocity are considered. Clearly, the step length serves to define the location (in the model) of the equivalent forces that are evaluated through Equations (1)–(5).

The selected pedestrian frequencies are reported in Table 9, together with the prevalent direction of the mode, the type of motion (walking or running), and the adopted damping ratio values.

The parameters adopted to model the pedestrian-induced load are given in Table 10 and Table 11. The values reported in Table 10 referring to Equation (1) concern the peak force ($G_{0}$), and $\alpha_{i}$ and $\varphi_{i}$ for the first three harmonics. In Table 11, for both walking (Equation (2)) and running (Equation (4)), the peak force ($F_{0}$), the factor k (which depends on the considered frequency), and the factor γ (which includes the dependence on damping), as well as the peak force ($G_{0}$) and the foot contact time ($t_{\mathrm{cr}}$) for the considered frequencies of 2.71 and 3.2 Hz in the running variants (1.7 and 1.8), are reported.

### 4.4. Pedestrian Frequency Dependence

Firstly, one pedestrian was considered to evaluate the deck acceleration for the eight investigated variants (Table 9), where the pedestrian-adopted models are those reported in Equation (1) for walking and Equation (3) for running. The considered path was Route 1 (Figure 7) and the adopted damping ratio values are those that were experimentally evaluated in 2019 [31]. Furthermore, depending on the frequency, they were found to be between 1.55 and 2.33%. The PAVs for the selected control points are summarized in Table 12.

The PAVs denote that the comfort level, according to the classification (Table 3) defined in SÉTRA [38], is (1) at a maximum along the horizontal lateral direction, since the $\mathrm{PAV}\;=0.14\;\frac{m}{s^{2}},$ which is lower than 0.15, (2) at a minimum along the vertical direction in terms of walking, since the $\mathrm{PAV}=1.93\;\frac{m}{s^{2}}\;$($f_{2}=1.64\;\mathrm{Hz}$) is between 1.0 and 2.5 $\frac{m}{s^{2}}$, and (3) uncomfortable along the vertical direction in terms of running ($f_{3}=2.71\;\mathrm{Hz}$), since the $\mathrm{PAV}=3.66\;\frac{m}{s^{2}}$, which is greater than 2.5 $\frac{m}{s^{2}}$.

#### 4.4.1. Multiple-Path Dependence

To evaluate the influence of different paths for walking, i.e., Variant 1.2, the highest value of the acceleration was attained and selected. The pedestrians were simulated through Equations (1)–(3).

The considered scenario is reported in Table 13, together with the peak acceleration values at each station. This was detailed as per the following: (1) the worst cases ($\mathrm{PAV}\approx 1.93\;\frac{m}{s^{2}}$) refer to the paths that include the central spans at station P3 (Routes 1, 3, and 4); (2) cases concerning those walking along the short spans (i.e., Route 2, and Routes 2 and 5), where the PAV achieved a value of 0.03 $\frac{m}{s^{2}}$ and which corresponds to ≈ 1.5 % of the worst case ($\approx 1.93\;\frac{m}{s^{2}}$); (3) when assuming the presence of three pedestrians, i.e., one pedestrian for each short span and one pedestrian for the middle span, a low difference in terms of PAV was obtained, whereby the central span was or was not excited in phase with the others.

#### 4.4.2. The Dynamic Response vs. the Damping Ratio

Preliminarily, it has to be recalled that for an SDOF system in resonance with a pulsating force with amplitude F, having a circular frequency $\omega_{D}$, stiffness K, and low value of the critical damping ξ, the normalized value of the displacement [49] and acceleration are given by Equations (11) and (12). Those expressions, which are normalized in terms of dividing by the static displacement $\frac{F}{K}$, include the transient and steady-state terms. It is worth noting that in the exponential term, the circular frequency has been substituted with $\frac{2\pi }{T}$ in order to express the solution in terms of the normalized time ($t^{*}$), defined herein, which is the current time divided by the period (T) of the SDOF.

(11) $$R_{D}\left(t\right)\approx \;\frac{1}{2\zeta }\left(e^{-2\pi \frac{t}{T}\xi }-1\right)cos\;2\pi \frac{t}{T}=\frac{1}{2\zeta }R^{*}\left(\xi ,t^{*}\right)\cos2\pi t^{*}$$

(12) $$R_{a}\left(t\right)=\omega_{D}^{2}\frac{1}{2\zeta }R_{a}^{*}\left(\xi ,t^{*}\right)\cos2\pi t^{*}\approx \omega_{D}^{2}\frac{1}{2\zeta }R^{*}\left(\xi ,t^{*}\right)\cos2\pi t^{*}$$

**Figure 12:**
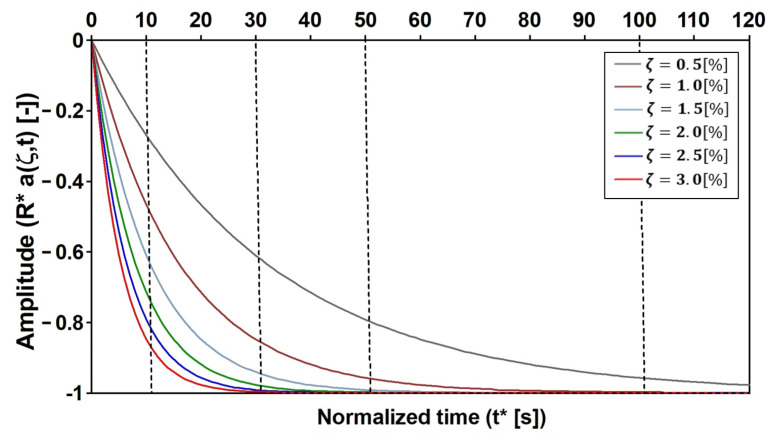
The SDOF when subjected to a harmonically varying load force, specifically, the natural conditions. The transient term, at resonance, vs. the normalized time and the critical damping.

It is usually supposed that the exponential term quickly vanishes and, consequently, that the magnification factor ($\frac{1}{2\xi }$) is inversely proportional to the damping ratio (ξ). However, these previous assumptions cannot be assumed to be general since they depend on both ξ and $t^{*}$. Furthermore, the transitory values can be considered negligible only if $R^{*}\left(\xi ,t^{*}\right)\to -1$. In addition, the greater $t^{*}$ is, the lower the dependence of $R^{*}$ on ξ (Figure 12).

**Figure 13:**
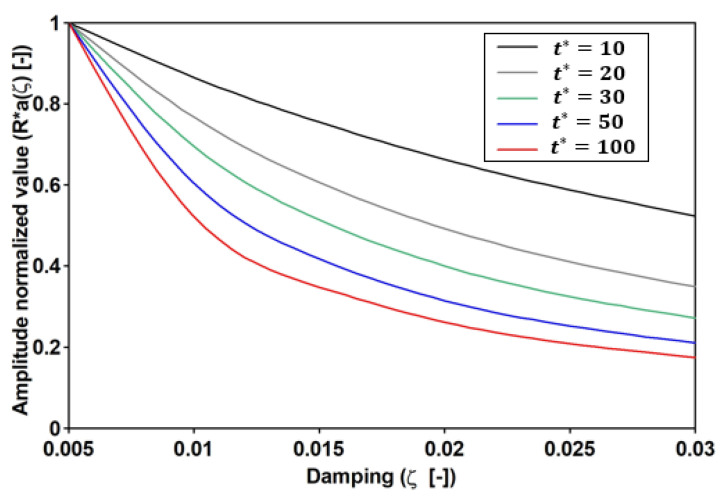
The SDOF that is subjected to a harmonically varying load force; specifically, the normalized general solution.

Further, the normalized general solution is reported in Figure 13, where the four values of $t^{*}$ were considered (10, 20, 30, and 50). For each of those values, in correspondence with the six considered critical damping ($\xi_{i}$), the $R_{a}^{*}=R^{*}$ values were evaluated to obtain the enveloping functions, i.e., $\frac{1}{2\xi }R_{a}^{*}\left(\xi \right)$. Furthermore, the function was evaluated at $t^{*}=100$, where $R_{a}^{*}\left(\zeta ,t^{*}\right)\to -1$. This is close to the steady-state solution and it can be argued that the lower the $t^{*}$, the greater the influence of the transitory values.

Based on this premise, the role of damping regarding pedestrian-induced vibrations was further investigated. Variants 1.1 and 1.2 (walking condition), and Variant 1.7 (running condition) were considered. For those variants, which are reported in Table 14, the accelerations were already evaluated (Table 12) using the damping values that were defined in 2019 [31]. This was conducted such that the influence of the damping could be further investigated, and was performed by using the values that were acquired in 2000 [27].

The obtained results are given in Table 14, where for each reported variant, there exists the following: (1) the frequency (f) of the imposed force, which is understood in resonance or is close to the pertinent mode; (2) the damping (ξ) value adopted for the resonant mode; (3) the ratio ($\frac{\zeta_{2019}}{\zeta_{2000}}$) of the damping value obtained from the 2019 and 2020 records; and (4) the peak acceleration (PAV) and their ratio ($\frac{{\mathrm{PAV}}_{2020}}{{\mathrm{PAV}}_{2019}}$) obtained from the 2020 and 2019 records. Furthermore, these results are based on previously discussed items, such that if the transitory is negligible, the magnification factor is equal to $\frac{1}{2\xi_{i}}$, and we should have $\frac{\xi_{2019}}{\zeta_{2020}}=\frac{{\mathrm{PAV}}_{2020}}{{\mathrm{PAV}}_{2019}}$. It is important to note the significance of the transitory conditions in the general solutions, since the values of $\frac{\zeta_{N}}{{\mathrm{PAV}}_{N}}$ range between 1.37 and 1.98. Through this, the importance of the transitory phase can be argued.

**Figure 14:**
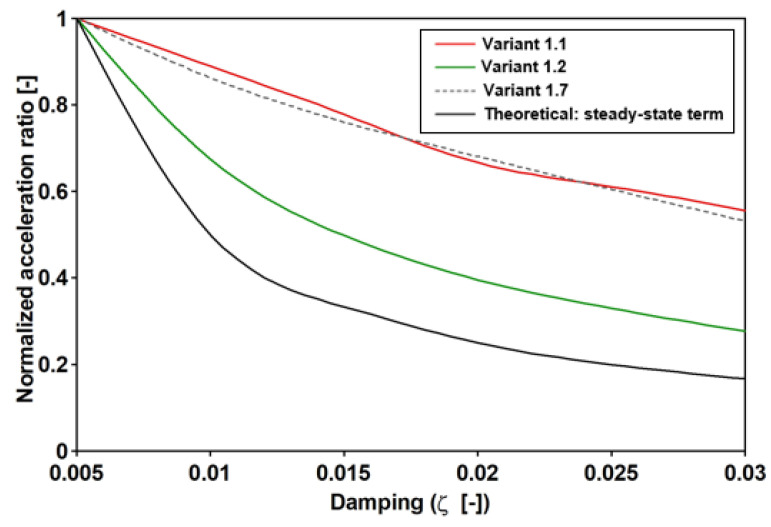
The sensitivity of the dynamic response vs. the damping of the footbridge for the lateral vibrations that are induced by walking ($f_{1}=1.88\;\mathrm{Hz}$), the vertical vibrations that are induced by walking ($f_{2}=1.64\;\mathrm{Hz}$) and the vertical vibrations that are induced by running ($f_{5}=2.71\;\mathrm{Hz}$).

Previous remarks were further checked by considering the six damping values ranging between 0.5 and 3.0%. Each value was assumed to be equal for each structural frequency, the results of which are reported in Table 15. Furthermore, in Figure 14, for each Variant, the normalized accelerations are reported together with the theoretical trend, ($\frac{1}{2\xi_{i}}$), which is to be expected for an SDOF when the transitory is negligible. Moreover, the normalization was conducted by dividing the acceleration by the PAV, which corresponded to ξ=0.5%.

**Figure 15:**
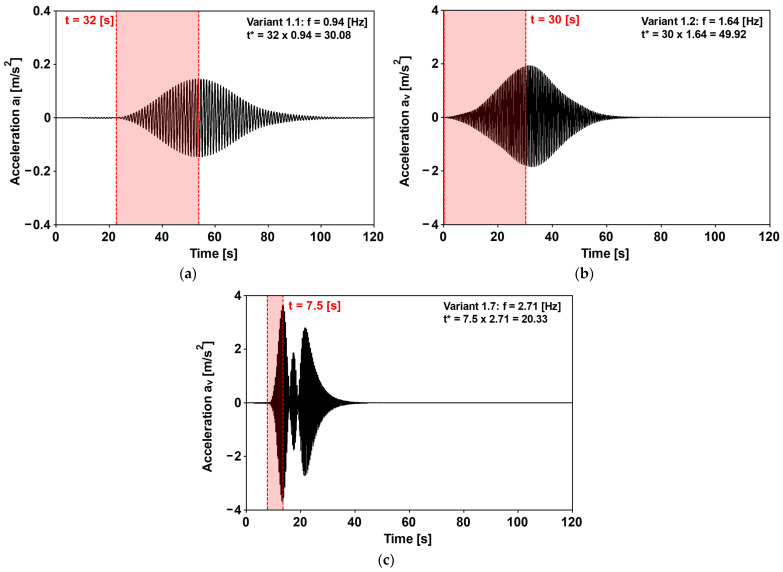
The evaluation of the normalized time for (**a**) Variant 1.1—the lateral vibrations that are induced by walking ($f_{1}=1.88\;\mathrm{Hz}$); (**b**) Variant 1.2—the vertical vibrations that are induced by walking ($f_{2}=1.64\;\mathrm{Hz}$); and (**c**) Variant 1.7—the vertical vibrations that are induced by running ($f_{5}=2.71\;\mathrm{Hz}$). The time histories at the P3 station are obtained by assuming the 2019 experimental critical damping values [31].

By matching the value reported in Figure 13 against those reported in Figure 14, it can be recognized that the Variant 1.2 solution could be attained at $t^{*}$ = 30, while variants 1.1 and 1.7 were attained at $t^{*}=10$. It is worth noting that the obtained values of $t^{*}$ concern an SDOF that is excited by a single frequency force. Meanwhile, the examined real cases concern an MDOF system that is differently excited. However, based on the obtained time histories (Figure 15), it follows that measuring $t^{*}$ considers the time between the beginning of the excitation and the PAV attainment, and that the obtained value of $t^{*}$ is different but not far from the analytical value. For instance, the analytical values of Variants 1.1 and 1.7 are 10, but the numerical values are 30 and 20, respectively, and, the analytical value of Variant 1.2 is 30, but the numerical value is 50.

### 4.5. Comfort Criteria Assessment vs. the Definition of a New Comfort Parameter

By having the time histories of the accelerations for the different scenarios, the comfort criteria assessment was pursued as per the following: (1) the pedestrian walking was modeled through Equations (1)–(3) (which was achieved by only considering the fundamental harmonic) and Equation (4); (2) the accelerations were evaluated at the five stations reported in Figure 9; and (3) the acceleration evaluated at each station was meant to be received by either the walking/running pedestrian or by a pedestrian at rest (it is worth noting that the previous differentiation does not affect the induced acceleration but rather the level of the acceptable limit [42]), which is reported in Figure 7 and Table 7.

#### 4.5.1. ISO 10137:2007(E) [42]

The RMS (Equation (7)) was evaluated by considering ∆t=1 s. The suggested threshold was obtained by amplifying the normalized function, as reported in Figure 9a (side-to-side) and Figure 9b (back-to-chest), and by the factor reported in Table 7 where the following are suggested: (1) the values of 30 and 60 are, respectively, for the standing and walking pedestrian when the bridge is subjected to vertical accelerations; and (2) a value of 60 for the horizontal situations of both standing and walking conditions.

**Figure 16:**
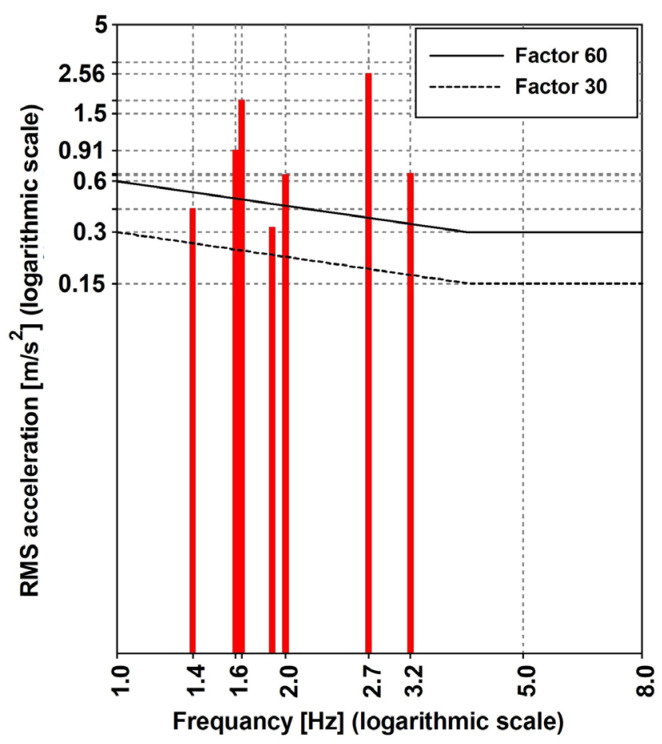
The ISO 10137:2007 [42] comfort criteria assessment for the vertical direction.

When considering the horizontal direction, the comfort criteria are fulfilled as follows: the RMS that is obtained for the walking condition is 0.13 $\frac{m}{s^{2}}$, which is lower than the threshold (0.216 $\frac{m}{s^{2}}$) for the frequency range (1–2 Hz). As far as the vertical direction is concerned, the evaluated RMS values are reported in Figure 16, together with the threshold that is suggested for the pedestrian at rest or when performing walking/running activities. For this, (1) the frequencies (Table 12) of the variants 1.2–1.6 (walking) and 1.7 (running) were considered, and (2) the factors equal to 30 (standing pedestrian) and 60 (walking and running) were also assumed. It is worth noting that the amplification factors (see Table 7) do not consider the running condition, and the assumed value of 60, is, according to the opinion of the authors, a conservative assumption, being the sensitivity of a runner, which is lower than the sensitivity of a walker.

It can be argued that the comfort limit was not respected, and the worst cases were found in the standing condition. As such, the following was considered: for the Variant 1.7—running, f=2.7 Hz, was achieved by predicting the $\mathrm{RMS}=2.56\;\frac{m}{s^{2}}$, and the limit value was $\approx 0.15\;\frac{m}{s^{2}}$; likewise, for the Variant 1.2—walking, f=1.64 Hz was achieved by predicting the $\mathrm{RMS}=1.79\;\frac{m}{s^{2}}$, and the limit value was between 0.15 and 0.30 $\frac{m}{s^{2}}$.

#### 4.5.2. EN 1990:2002+A1 [37] and BS EN 1991-2:2003 [43]

According to Eurocode 1 [37], the acceleration peak value (PAVs) for vertical accelerations is 0.7 $\frac{m}{s^{2}}$. Meanwhile, the British Standard [43] suggests evaluating the threshold according to Equation 5, where, assuming for $k_{i}$, the value that is reported in Table 6 gives a limit of 1.46 $\frac{m}{s^{2}}$. It then follows (see Table 16) that according to Eurocode 1, the limit is respected only for load variants 1.4 and 1.6, and, according to the British Standard, the limit is not fulfilled for Variant 1.2.

#### 4.5.3. SÉTRA [38] Technical Guide

**Figure 17:**
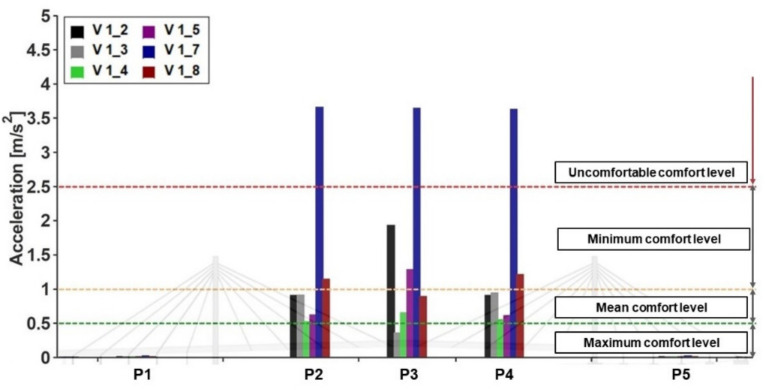
The comfort criteria assessment along the span of the bridge (as per SÉTRA [36]).

**Figure 18:**
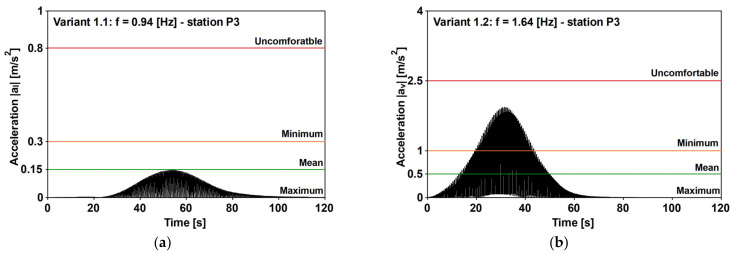

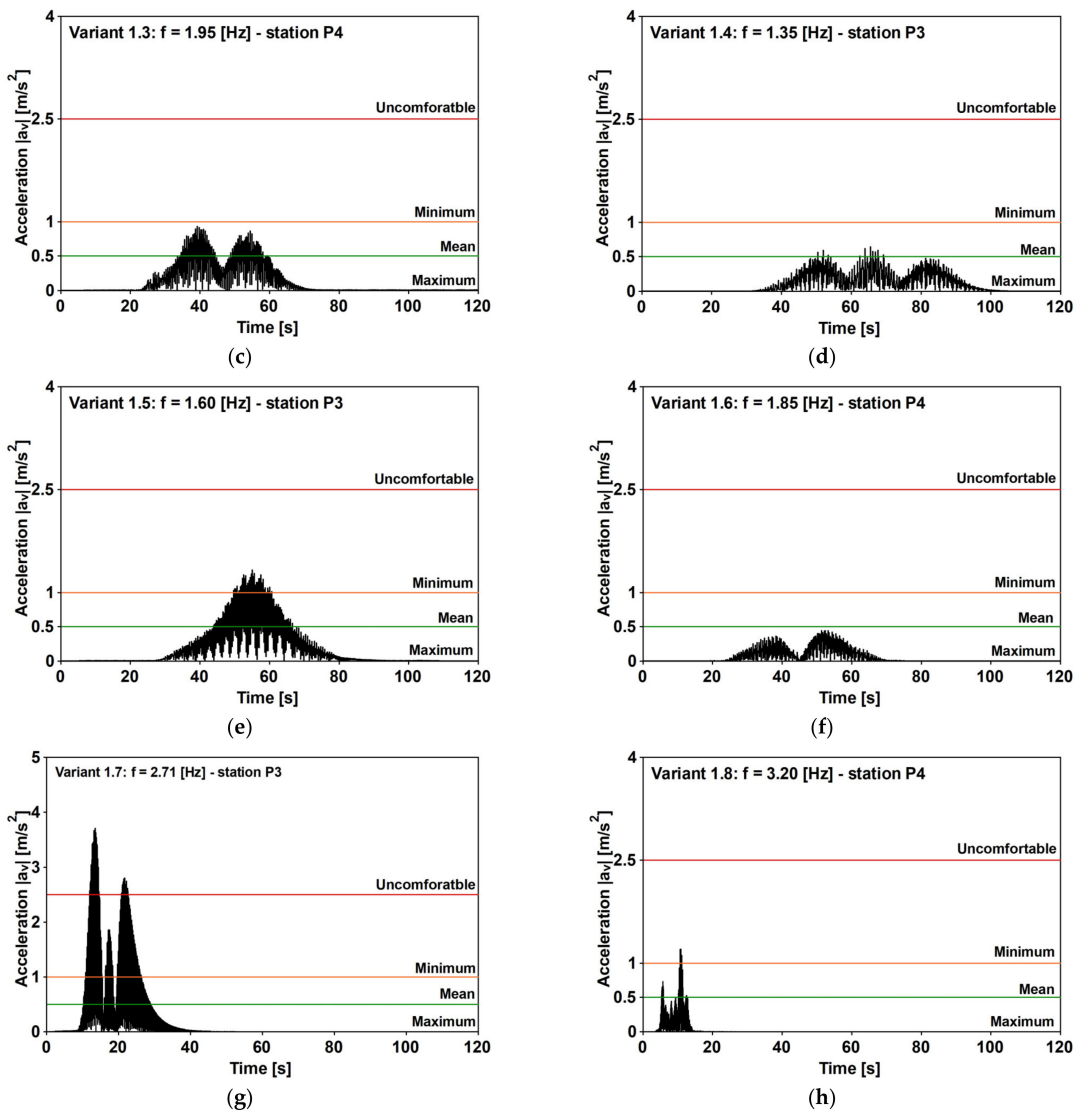
The absolute acceleration values vs. the SÉTRA [36] comfort level. The walking (**a**–**f**) and running (**g**,**h**) simulations are according to the considered load variants (see Table 9).

When the horizontal condition is considered, the PAV (Table 12) was 0.14 $\frac{m}{s^{2}}$, such that the comfort level was the maximum (Table 3). As far as the vertical direction was considered, the obtained results for this are reported in Figure 17 and Figure 18, together with the recommended thresholds. The values reported in Figure 17 relate to the peaks recorded at stations P2, P3, and P4, which are located (see Figure 7) along the central span. Meanwhile, those reported in Figure 18 relate to the absolute values of the time histories at the stations where the PAVs were higher. Furthermore, it can be argued that the footbridge meets the requirements of the minimum comfort level for all of the walking conditions, and for the running condition (Variant 1.7, 2.71 Hz), the uncomfortable situation is triggered. Further still, it can be noted that when a threshold is not respected (Figure 18), the lower the comfort level, the shorter the period corresponding to the given level not being fulfilled. As such, according to the authors, the different levels of comfort should include a parameter that depends on the amount of time the pedestrians spend at a given level of discomfort.

## 5. Conclusions

In general, for the Aberfeldy footbridge, the dynamic comfort rating can be specified as the minimum. Considering the location and all of the objectives for using the examined structure, satisfactory conditions were achieved for vibration serviceability. However, from this study, the following main remarks can be provided:

- The numerical modal evaluation showed that the two fundamental natural frequencies of the Aberfeldy footbridge were in a range that corresponds with a maximum risk of resonance. It is worth noting that the risk of resonance for the investigated footbridge, in terms of the human-induced dynamic loading, could be more significant than for other types of composite material footbridges, i.e., double-tied or U-beam structures [21];
- The response of this structure to pedestrian-induced dynamic loading with the frequency around eigenvalues was evaluated. For the lateral direction, comfort criteria passed the criteria of the SÉTRA document [38], EN 1990:2002+A1 [37], BS EN 1991-2:2003 [43], and ISO 10137:2007 (E) [42]. Meanwhile, for the vertical direction, the limits that were exceeded were identified;
- The second harmonic of the dynamic forces that were generated by a typical pedestrian walking across the bridge affects the comfortable use of the footbridge. The recommendation that the frequency limits for light footbridges should be extended to account for excitation by higher harmonics [24] has found justification in respect of the Aberfeldy footbridge;
- Since the Aberfeldy footbridge has three spans, multiple-path dependence was considered. It was concluded that the part of the structure most susceptible to human-induced dynamic excitation was the middle span of the bridge;
- It appears that an additional procedure to assess the comfort criteria assessment for light footbridges is required to quantify the time that a pedestrian spends at a given level of discomfort.

## Figures and Tables

**Table 1:** Monitoring activities: the frequencies and related damping ratios.

Mode *	Frequency [Hz]			Damping Ratio [%]		
	1995 [26]	2000 [27]	2019 [31]	1995 [26]	2000 [27]	2019 [31]
H1	1.00	0.98	0.95	-	1.0	1.56
V1	1.59	1.52	1.53	0.84	0.4	1.83
V2	1.92	1.86	1.84	0.94	0.7	2.33
V3	2.59	2.49	2.48	1.20	0.7	1.50
H2	2.81	2.73	2.63	-	1.2	2.14
V4	3.14	3.01	-	-	0.8	-
T1	3.44	3.48	-	-	5.5	-
V5	3.63	3.50	3.46	-	0.6	2.37
V6	4.00	3.91	3.84	-	0.9	4.63
T2	4.31	4.29	-	-	3.2	-
V7	4.60	4.40	-	-	0.8	-
V8	5.10	4.93	4.91	-	1.8	2.40

* H = horizontal; V = vertical; and T = torsional.

**Table 2:** The frequency, velocity, and stride length range for walking, jogging, and running [35,36].

Type of Motion	Frequency Ranges $f_{u}$ [Hz]	Velocity $v_{u}$ [m/s]	Stride Length $l_{u}$ [m]
Slow walking	~1.7	1.1	0.60
Normal walking	~2.0	1.5	0.75
Fast walking	~2.3	2.2	1.00
Slow running	~2.5	3.3	1.30
Fast running	>2.3	5.5	1.75

**Table 3:** The resonance risk levels and corresponding ranges of the natural frequencies [38].

Risk of Resonance	Frequency Ranges Hz	
	Vertical	Horizontal
Maximum	1.7–2.1	0.5–1.1
Medium	1.0–1.7; 2.1–2.6	0.3–0.5; 1.1–1.3
Low	2.6–5.0	1.3–2.5
Negligible	0–1.0; >5.0	0–0.3; >2.5

**Table 4:** The considered paths and pedestrian numbers.

Paths	No. of Pedestrians
I—Route no. 1	1
II—Route no. 2	1
III—Route no. 3	1
IV—Route no. 4	1
V—Routes no. 2 and 5	1 + 1
VI—Routes no. 2, 4, and 5, in phase	1 + 1 + 1
VII—Routes no. 2, 4, and 5, counterphase	1 + 1 + 1

**Table 5:** The acceleration ranges associated with comfort levels (SÉTRA [36]).

Comfort Level	Ranges of Comfort [$\frac{m}{s^{2}}$]	
	Vertical	Horizontal
Maximum	0.0–0.5	0.00–0.15
Mean	0.5–1.0	0.15–0.30
Minimum	1.0–2.5	0.30–0.80
Uncomfortable	>2.5	>0.8

**Table 6:** The Aberfeldy footbridge serviceability limits for vertical acceleration, based on the UK National Annex to Eurocode 1 [43].

Parameters	Assumable Values	Recommended Serviceability Limits for Acceleration (Equation (10))
Bridge function	$k_{1}=1.6$—for rural environments	$a_{limit\;}=1.46\;\frac{m}{s^{2}}$
Route redundancy	$k_{2}=1.3$—for alternative routes readily available	
Bridge height	$k_{3}=0.7$—for structural heights > 8 m	
Exposure	$k_{4}=1.0$—for normal exposure	

**Table 7:** The multiplying factors used to specify the satisfactory magnitudes of footbridge vibration with respect to pedestrian responses (ISO [42]).

Direction of Vibration	Pedestrian Scenario	Multiplying Factor
Vertical	Standing	30
Vertical	Walking	60
Horizontal	Standing or walking	60

**Table 8:** The element and material properties of the lamina.

$E_{11}\left[GPa\right]$	$E_{22}\left[GPa\right]$	$G_{12}\left[GPa\right]$	$G_{23}\left[GPa\right]$	$\nu_{21}\left[-\right]$	$\nu_{21}\left[-\right]$
22.00	6.77	2.72	1.67	0.19	0.058

**Table 9:** The basic load variants vs. the modal parameters of the footbridge. The adopted damping values are those defined in 2019 [31].

No. of Load Variant	FE Natural Frequency of Footbridge [Hz]	Mode	Damping [%]	Frequency of Loading [Hz]	Step Length	Type of Motion
1.1	0.94	H1	1.56	0.94	0.70	Walking
1.2	1.64	V1	1.83	1.64	0.60	
1.3	1.95	V2	2.33	1.95	0.70	
1.4	2.71	V3	1.50	1.35	0.60	
1.5	3.20	V4	-	1.60	0.60	
1.6	3.71	V5	2.37	1.85	0.70	
1.7	2.71	V3	1.50	2.71	1.20	Running
1.8	3.20	V4	-	3.20	1.40	

**Table 10:** The human-induced dynamic load (walking and running). Equations (2) and (3) parameter values.

Type of Motion	Direction	Parameter (Equations (2) and (3))						
		$G_{0}\;\left[kN\right]$	$\alpha_{1}$	$\alpha_{2}$	$\alpha_{3}$	$\varphi_{1}$	$\varphi_{2}$	$\varphi_{3}$
Walking	Vertical	0.70	0.37	0.10	0.12	0	π/2	π/2
	Lateral	0.70	0.1	0.0	0.1	0	π/2	π/2
Running*	Vertical	700	1.45	0.15	0.05	0	0	0

* Lateral direction was not considered for running.

**Table 11:** The human-induced dynamic load. Equation (2) (walking) and Equation (5) (running) parameter values.

Direction	Parameter (Equation (2))			Parameter (Equation (3))		
	$F_{0}\;\left[kN\right]$	$k\left(f_{v}\right)$	γ	$G_{0}\;\left[kN\right]$	$t_{cr\left(2.71Hz\right)}$	$t_{cr\left(3.20Hz\right)}$
Vertical	0.28	1	0.225	0.70	0.23	0.21

**Table 12:** The dynamic response of the footbridge for a one-pedestrian passage (PAVs).

Load Variant	Scenario	PAVs $\;\left[\frac{m}{s^{2}}\right]$				
		P1	P2	P3	P4	P5
1.1 (lateral)	Walking (f=1.88 Hz)	0.00	0.08	0.14	0.08	0.00
1.2 (vertical)	Walking (f=1.64 Hz)	0.01	0.90	1.93	0.90	0.01
1.3 (vertical)	Walking (f=1.95 Hz)	0.01	0.91	0.36	0.94	0.01
1.4 (vertical)	Walking (f=1.35 Hz)	0.01	0.52	0.65	0.55	0.01
1.5 (vertical)	Walking (f=1.60 Hz)	0.01	0.62	1.28	0.61	0.01
1.6 (vertical)	Walking (f=1.85 Hz)	0.01	0.45	0.39	0.46	0.01
1.7 (vertical)	Running (f=2.71 Hz)	0.02	3.66	3.64	3.63	0.02
1.8 (vertical)	Running (f=3.20 Hz)	0.01	1.14	0.89	1.21	0.01

**Table 13:** The dynamic response of the footbridge for the different configurations of routes for Load Variant 1.2 (f=1.64 Hz).

Scenario	PAVs $\;\left[\frac{m}{s^{2}}\right]$				
	P1	P2	P3	P4	P5
No. of pedestrians: 1; Route no. 1	0.01	0.90	1.93	0.90	0.01
No. of pedestrians: 1; Route no. 2	0.00	0.01	0.02	0.01	0.00
No. of pedestrians: 1; Route no. 3	0.01	0.90	1.93	0.90	0.01
No. of pedestrians: 1; Route no. 4	0.01	0.89	1.92	0.88	0.01
No. of pedestrians: 2; Routes 2 and 5	0.00	0.02	0.03	0.02	0.00
No. of pedestrians: 3; Routes 2, 4, and 5; in phase	0.01	0.88	1.91	0.89	0.01
No. of pedestrians: 3; Routes 2 and 5, as well as 4 in counterphase	0.02	0.90	1.94	0.91	0.02

**Table 14:** Peak acceleration (PAV at the P3 station) vs. the frequency-dependent damping.

	Variant 1.1			Variant 1.2			Variant 1.7		
	$f\;\left[Hz\right]$	$\xi \;\left[\%\right]$	$PAVs\;\left[\frac{m}{s^{2}}\right]$	$f\;\left[Hz\right]$	$\xi \;\left[\%\right]$	$PAVs\;\left[\frac{m}{s^{2}}\right]$	$f\;\left[Hz\right]$	$\xi \left[\%\right]$	$PAVs\;\left[\frac{m}{s^{2}}\right]$
2019 [31]	0.94	1.56	0.14	1.64	1.83	1.93	2.7	1.50%	3.64
2000 [27]		1.00	0.16		0.40	5.01		0.70	3.95
$\xi_{N}=\frac{\zeta_{2019}}{\zeta_{2000}}$		1.56			4.57			2.14	
${\mathrm{PAV}}_{N}=\frac{{\mathrm{PAV}}_{2000}}{{\mathrm{PAV}}_{2019}}$		1.14			2.64			1.08	
$\frac{\xi }{{\mathrm{PAV}}_{N}}$		1.37			1.73			1.98	

**Table 15:** Peak acceleration (PAV at the P3 station) vs. the frequency-independent damping (ζ).

Damping	PAVs$\;\left[\frac{m}{s^{2}}\right]$ at Point P3 vs. Damping [%]					
	0.5	1.0	1.5	2.0	2.5	3.0
PAV: Variant 1.1	0.18	0.16	0.14	0.12	0.11	0.10
PAV: Variant 1.2	4.52	3.05	2.25	1.79	1.49	1.25
PAV: Variant 1.7	4.88	4.21	3.71	3.32	2.95	2.59

**Table 16:** The EN 1990:2002+A1 [37] and BS EN 1991-2:2003 [43] comfort criteria assessments.

Model	$PAVs\;\left[\frac{m}{s^{2}}\right]$ vs. the Load Variants (Frequency [Hz])				
	1.2 (1.64)	1.3 (1.95)	1.4 (1.35)	1.5 (1.60)	1.6 (1.85)
Equations (1)–(3)	1.93	0.94	0.65	1.28	0.46
Equation (4)	2.20	1.21	0.24	1.52	0.51

## Data Availability

Not applicable.

## References

[1] Capozucca R., Magagnini E., Bettucci E. (2022). Delamination buckling of GFRP-strips in strengthened RC beams. Compos. Struct..

[2] Purwanto E., Adri P.A., Kristiawan S.A., Sangadji S., Halwan S.H. (2022). Strengthening of non-engineered building beam-column joint to increase seismic performance with variation of steel plate width. Lecture Notes in Civil Engineering, Proceedings of the 5th International Conference on Rehabilitation and Maintenance in Civil Engineering: ICRMCE 2021, Surakarta, Indonesia, 8–9 July 2021.

[3] Gatesco N. (2011). New Materials for the Rehabilitation of Cultural Heritage.

[4] Bank L.C. (2006). Application of FRP composites to bridges in the USA. Proceedings of the International Colloquium on Application of FRP to Bridges.

[5] Hollaway L.C. (2010). A review of the present and the future utilization of FRP composites in the civil infrastructure with reference to their important in-service properties. Constr. Build Mater..

[6] Pyrzkowski Ł., Misiewicz M. Modern GFRP Composite Footbridges. Proceedings of the 10th International Conference “Environmental Engineering”.

[7] Smits J. (2016). Fiber-Reinforced Polymer Bridge Design in the Netherlands: Architectural Challenges toward Innovative, Sustainable, and Durable Bridges. Engineering.

[8] Alper H., Barton F.W., McCormick F.C. (1977). Optimum design of a reinforced plastic bridge girder. Comput. Struct..

[9] Gao H., Sun Y., Jian J., Dong Y., Liu H. (2023). Study on mechanical properties and application in communication pole line engineering of glass fiber reinforced polyurethane composites (GFRP). Case Stud. Constr. Mater..

[10] Stankiewicz B., Tatara M. (2015). Applications of glass and glass fiber retrofit polymer in modern footbridges. J. Civil Eng. Archit..

[11] Quadrino A., Damiani M., Penna R., Feo L., Nisticò N. (2022). Lecture Notes in Civil Engineering.

[12] Areiza-Hurtado M., Bansal A., Paulotto C., Primi S. FRP girder bridges: Lessons learned in Spain in the last decade. Proceedings of the 6th International Conference on FRP Composites in Civil Engineering (CICE).

[13] Górriz P., Bansal A., Paulotto C., Primi S., Calvo I. (2017). Composite Solutions for Construction Sector. Case Study of Innovative Projects—Successful Real Cases.

[14] Stankiewicz B. (2015). Bridge structures with GFRP composite deck. Open J. Civ. Eng..

[15] Burgoyne C.J., Head P.R. Aberfeldy Bridge—An advanced textile reinforced footbridge. Proceedings of the Techtextil Symposium.

[16] Sobrino J.A., Pulido M.D.G. (2002). Towards Advanced Composite Material Footbridges. Struct. Eng. Int..

[17] Keller T., Bai Y., Vallée T. (2007). Long-Term Performance of a Glass Fiber-Reinforced Polymer Truss Bridge. J. Compos. Constr..

[18] Adilardi A., Frasconi L. Design of a pedestrian bridge made with pultruded profiles of fibreglass-reinforced plastics in Prato. Proceedings of the 3rd International Conference on Footbridges.

[19] Votsis R.A., Stratford T.J., Chryssanthopoulos M.K., Tantele E.A. (2017). Dynamic assessment of a FRP suspension footbridge through field testing and finite element modelling. Steel Compos. Struct..

[20] Górski P., Stankiewicz B., Tatara M. (2017). Modal parameter identification of all-GFRP composite cable-stayed footbridge in Denmark Case. Proceedings of the Dynamics of Civil Engineering and Transport Structures and Wind Engineering—DYN-WIND’2017, MATEC Web of Conferences.

[21] Wei X., Boscato G., Russell J., Adilardi A., Russo S., Živanović S. (2019). Experimental Characterisation of Dynamic Properties of an All-FRP Truss Bridge. Dynamics of Civil Structures, Volume 2: Proceedings of the 36th IMAC, A Conference and Exposition on Structural Dynamics 2018.

[22] Drygala I.J., Polak M.A., Dulinska J.M. (2019). Vibration serviceability assessment of GFRP pedestrian bridges. Eng. Struct..

[23] Drygala I.J., Dulinska J.M., Ciura R., Lachawiec K. (2020). Vibration Serviceability of Footbridges: Classical vs. Innovative Material Solutions for Deck Slabs. Materials.

[24] Wei X., Russell J., Živanović S., Mottram J.T. (2019). Measured dynamic properties for FRP footbridges and their critical comparison against structures made of conventional construction materials. Compos. Struct..

[25] Russell J.M., Wei X., Živanović S., Kruger C. (2020). Vibration serviceability of a GFRP railway crossing due to pedestrians and train excitation. Eng. Struct..

[26] Gallegos-Calderón C., Renedo C.M., Pulido M.D.G., Díaz I.M. (2022). A frequency-domain procedure to design TMDs for lively pedestrian structures considering Human–Structure Interaction. Structure.

[27] Pimentel R.L., Waldron P., Harvey W.J. (1995). Assessment of the dynamic behaviour of Aberfeldy GRP plastic cable-stayed footbridge. Proceedings of the Analysis & Testing of Bridges: Papers Presented at A One Day Seminar.

[28] Pavic A., Reynolds P., Cooper P., Harvey W.J. (2000). Dynamic Testing and Analysis of Aberfeldy Footbridge.

[29] Cadei J., Stratford T. (2002). The design, construction and in-service performance of the all-composite Aberfeldy footbridge. Advanced Polymer Composites for Structural Applications in Construction: Proceedings of the First International Conference, Held at Southampton University, Southampton, UK, 15–17 April 2002.

[30] Stratford T. The condition of the Aberfeldy Footbridge after 20 years of service. Proceedings of the Structural Faults and Repair 2012.

[31] Skinner J.M. A Critical Analysis of the Aberfeldy Footbridge, Scotland. Proceedings of the Bridge Engineering 2 Conference.

[32] Wynne Z., Stratford T., Reynolds T.P.S. (2021). Operational Modal Analysis of a Historic GRP Structure. Lecture Notes in Civil Engineering, Civil Structural Health Monitoring, Proceedings of CSHM-8 Workshop, Naples, Italy, 31 March–2 April 2021.

[33] Burgoyne C.J. (1987). Structural use of Parafil ropes. Constr. Build. Mater..

[34] Burgoyne C.J. (1993). Parafil ropes for prestressing applications. Fiber-Reinforced-Plastic (FRP) Reinforcement for Concrete Structures: Properties and Applications.

[35] Damiani M., Quadrino A., Nisticò N. (2021). FRP Cables to Prestress RC Beams: State of the Art vs. a Split Wedge Anchorage System. Buildings.

[36] Wheeler J.E. (1982). Prediction and control of pedestrian induced vibration in footbridges. J. Struct. Div-ASCE..

[37] Zivanovic S., Pavic A., Reynolds P. (2005). Vibration serviceability of footbridges under human-induced excitation: A literature review. J. Sound Vib..

[38] (2005). Eurocode—Basis of Structural Design.

[39] Sétra F. (2006). Assessment of Vibrational Behaviour of Footbridges under Pedestrian Loading, Technical Guide.

[40] Tongen A., Wunderlich R.E. (1994). Biomechanics of running and walking. Clin. Sport. Med..

[41] Bachmann H., Ammann W., Deischl F., Eisenmann J., Floegl I., Hirsch G.H., Klein G.K., Lande G.J., Mahrenholtz O., Natke H.G. (1995). Vibration Problems in Structures: Practical Guideline.

[42] Bachmann H., Pretlove A.J., Rainer H. (1995). Dynamic forces from rhythmical human body motions. Vibration Problems in Structures: Practical Guidelines, Appendix G..

[43] (2007). Bases for design of structures—Serviceability of buildings and walkways against vibrations.

[44] (2003). UK National Annex to Eurocode 1, Actions on structures, Traffic loads on bridges.

[45] Occhiuzzi A., Spizzuoco M., Ricciardelli F. (2008). Loading models and response control of footbridges excited by running pedestrians. Struct. Control Health Monit..

[46] Marecik K., Pańtak M. (2018). A comparative analysis of selected models of pedestrian-generated dynamic loads on footbridges-vertical loads. Proceedings of the 3rd International Workshop on Flexibility in Sustainable Construction (ORSDCE 2018), MATEC Web of Conferences.

[47] Pańtak M. (2020). Ground Reaction Forces Generated by Runners—Harmonic Analyses and Modelling. Appl. Sci..

[48] Abaqus S. (2015). Users’ Manual Version 6.13 Documentation, c2013–2015.

[49] Jones R.B. (2018). Mechanics of Composite Materials.

